# Microcracking pattern in fractured bones: new approach for distinguishing between peri- and postmortem fractures

**DOI:** 10.1007/s00414-022-02875-1

**Published:** 2022-09-06

**Authors:** Michelle Winter-Buchwalder, Nathalie Schwab, Ignasi Galtés, Marisa Ortega-Sánchez, Sarah Scheirs, Xavier Jordana

**Affiliations:** 1 Faculty of Life Sciences, Department of Molecular Biology, University of Vienna, Dr. Bohrgasse 9, 1030 Vienna, Austria; 2 Forensic Anthropology Unit, Institut de Medicina Legal I Ciènces Forenses de Catalunya (IMLCFC), Ciutat de la Justícia, Gran Via de les Corts Catalanes, 111 Edifici G, 08075 Barcelona, Spain; 3 Research Group of Biological Anthropology (GREAB), Biological Anthropology Unit, BABVE Department, Universitat Autònoma de Barcelona, Cerdanyola del Vallès, 08193 Barcelona, Spain; 4 Legal Medicine Unit, Department of Psychiatry and Legal Medicine, Universitat Autònoma de Barcelona, Cerdanyola del Vallès, 08193 Barcelona, Spain; 5 Anatomy and Embryology Unit, Morphological Sciences, Faculty of Medicine, Autonomous Universitat Autònoma de Barcelona, Cerdanyola del Vallès, 08193 Barcelona, Spain; 6 The Tissue Repair and Regeneration Laboratory (TR2Lab), Experimental Sciences and Methodology Department, Faculty of Health Science and Welfare, University of Vic – Central University of Catalonia, Sagrada Família 7, Vic, 08500 Barcelona, Spain

**Keywords:** Blunt force trauma, Fracture timing, Bone histology, Forensic anthropology

## Abstract

Timing bone fractures is one of the main tasks of a forensic anthropologist, but still an uncertain diagnostic. In the literature, there are many macroscopic methods to distinguish perimortem from postmortem fractures, based on the distinct structural and mechanical properties of fresh and dry bones. However, this differentiation is still challenging, in particular when the bones are fragmented or still exhibit fresh properties. Although histologic analysis is often used as a complementary diagnostic tool in forensic pathology, its application in the evaluation of bone fractures is uncommon. The aim of this study was to investigate whether fractures of fresh bones reveal a distinct microcracking pattern compared to fractures of dry bones, in order to optimise the fracture timing. To this purpose, we histologically analysed perimortem and postmortem fractures in human humeri. The fresh bones were retrieved from traumatic autopsy cases, and the dry bones from donors which were experimentally fractured. Our results showed that the highest density and length of microcracks (MCKs) were found in the interstitial area of dry fractured bones, which may be considered a marker of postmortem damage. In fresh fractured bones, we generally observed a lower density of MCKs, but a higher proportion of osteonal MCKs, which may be considered a marker of perimortem trauma. In summary, the results of our exploratory study suggest that changes in intrinsic bone factors (mineral/organic components) result in a different microcracking pattern that can be used in fracture timing.

## Introduction

Timing bone injury remains challenging during the assessment of skeletal remains. This analysis implies a distinction between antemortem, perimortem and postmortem [1–4]. Antemortem trauma occurs prior to death and reveals bone remodelling as a sign of healing. On the contrary, such evidence of healing is not present in both perimortem and postmortem fractures. A reliable distinction between perimortem trauma and postmortem damage is crucial, since the former may allow conclusions on the circumstances of death. Forensic anthropologists use the term perimortem to describe all injuries occurring to wet/fresh bone, when the bone still contains its organic components, despite the fact that somatic death may have already occurred [5]. In contrast, postmortem damage occurs when the bone has lost most of its organic elements, causing it to fracture like a dry bone. It implies the involvement of taphonomic factors such as geological, biological or (un)intentional human alterations [1–5]. The terms ante-, peri- and postmortem are used in a different way in the field of forensic pathology, where they are only used to define the time interval related to somatic death, and not bone degradation [6].

In the literature, there are many methodologies to establish the differential diagnosis between perimortem and postmortem fractures. These methods are based on the distinct structural and mechanical properties of fresh and dry bones. They include macroscopic criteria such as the colour, the smoothness or roughness of the fractured edges [2, 4, 7, 8]. Nevertheless, a reliable differentiation between perimortem trauma and postmortem damage is still challenging and needs further knowledge on distinctive fracture patterns, which may be acquired from complementary methods.

Studies focusing on bone histology to distinguish between perimortem and postmortem bone fractures are very scarce and inconclusive. Hanaue et al. [9] investigated osteon fracture patterns by performing sagittal splitting osteotomy in two fresh human mandibles. The authors observed that fracture lines use to run along the curve of the osteonal lamellae. In contrast, Pechníková et al. [10] analysed undecalcified sections of fracture margins after blunt force trauma in fresh and dry human long bones and found that the fracture line usually crosses the osteons rather than the lamellar systems. Interestingly, they did not find significant differences in the osteon fracture pattern between fresh and dry specimens. Ebacher et al. [11] examined the structure–microcracking relations in human cortical bone tissue following compressive loading. The authors described the presence of a distinct microcracking pattern within the lamellar structure of osteons. They showed that intrinsic factors such as bone composition and microstructure as well as extrinsic factors mainly implying trauma circumstances determine the microcrack (MCK) production and distribution. However, how the different structural levels of cortical bone are involved in the fracture process is still poorly understood.

When bone is subjected to blunt trauma, it will undergo two mechanisms before a fracture occurs. In the first phase, the bone will undergo elastic deformation. This means that the bone can return to its original dimensions. In the second phase, it will experience plastic deformation, which will deform the bone permanently. The plastic phase will continue until breaking occurs [5]. Fresh bone is moist and contains water and organic components such as lipids and collagen, which make the bone stiff and elastic. Dry bone lacks these organic components, causing the bone to become stiff and brittle [12]. The loss of viscoelasticity in dry bone increases the impact sensitivity, because it makes the bone unable to resist as much strain or elastic deformation as in wet bone. This causes dry bone to fracture immediately after the strength threshold is reached [1, 5]. Due to these distinct properties, the macroscopic fracture patterns in dry and fresh bones are different. Thus, we expect that the analysis of the histological microcracking pattern will also reveal differences.

In this basic and exploratory research, we approach the hypothesis that there is a distinct microcracking pattern related to blunt force trauma in fresh bones that allows us to optimise the distinction between perimortem and postmortem fractures. An enhanced understanding about microcracking patterns in perimortem bone trauma is potentially valuable for timing and reconstructing traumatic events in the forensic context.

## Material and methods

### Samples

This study used both perimortem (fresh) fractured specimens and healthy unfractured dry bone samples.

To assess the perimortem (fresh) microcracking pattern, five fractured fresh humeri from five traumatic death cases were used with a postmortem time interval (PMI) between 12 and 24 h. All cases presented butterfly fractures of the humeral shaft. The victims were involved in vehicle accidents or falls and came from forensic autopsies at the Institute for Legal Medicine and Forensic Science of Catalonia (IMLCFC), which were removed for complementary medicolegal investigation. In order to correctly compare the autopsy samples with the dry bone samples, only cases with victims aged 55 or older were included in the study.

For experimental purposes, six healthy and unfractured dry humeri were collected from adults (> 60 years) that donated their body to science. The donor bones were provided with a PMI of 15 up to 20 years by the Medical Anatomy Department of the Universitat Autònoma de Barcelona (UAB). This study was approved by the Ethic Commission of Human and Animal Experimental Work (CEEAH) of the UAB, in compliance with the ethical regulations.

### Fracture reproduction

The shaft of five out of six dry humeri was experimentally fractured. The experimental fractures were induced using a pendulum impact test machine consisting of a metal frame and a pendulum to which a hammer of 5 kg was attached [13]. A piece of soft rubber was attached to the hammer to avoid direct contact with the bone. The humeri were placed horizontally with the anterior side facing the hammer and attached with tie-wraps to two movable metal holders. The hammer hit perpendicularly the anterior side of the humeral shaft. All of them presented comminuted fracture.

### Bone preparation and histological analysis

The resultant dry fractures were histologically compared to the fresh fractures from the autopsy cases. To this purpose, autopsy specimens were previously defleshed and cleaned for a better fracture examination. The samples were cooked in a water detergent solution at 90–100 °C during 2 to5 h. If present, remaining soft tissue was removed using surgical tools and the bones were cleaned with water and left to dry. The dry samples were only cleaned with a soft brush.

**Fig. 1:**
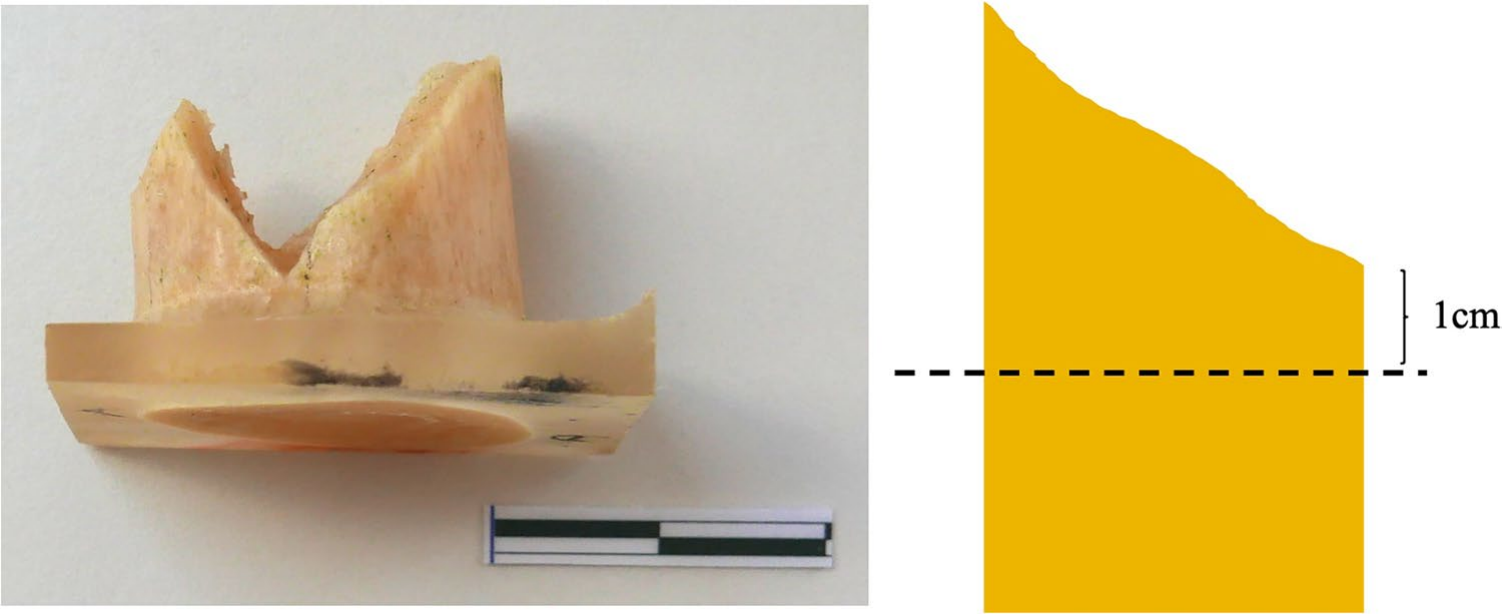
Bone fragment of a fractured fresh humerus embedded in epoxy resin and sectioned in a transverse plane 1 cm below the main fracture line. From the inferior side of the resin blocks, ground and polished thin sections of about 100-µm thickness were produced

For bone histological examination purpose, specimens were cut with an oscillating saw in blocks of 4 cm including the fractured region. Each block was fixated and dehydrated following the protocol by Ebacher et al. [11] and de Boer et al. [14]. The samples were sequentially kept in a series of ethanol solutions (70%, 80%, 90%, 100%) for 24 h per step. After being left to dry, the bone fragments were embedded in epoxy resin and sectioned in a transverse plane 1 cm below the main fracture line (Fig. 1), ground, polished and mounted on frosted glasses.

Thin sections were cut from the fixed bone blocks using a Buehler Isomet saw with a final thickness of 100 µm. Subsequently, the histological sections were put into an alcohol gradient (70%, 96%, 100%) followed by a cleaning agent (Histolemon®) to help fixing the section. Afterwards, DPX (Dibutylphthalate Polystyrene Xylene) was used as a mounting medium to bond the coverslip to the slide until it was polymerised.

To assure that our histological procedure does not produce a significant amount of MCKs, one of the dry humeri without fracture was analysed as a control. The fact that the dry condition makes the bone brittle and more sensitive to trauma allows us to test the effects of the process with a high accuracy. Histological inspection of the cortical bone was performed with a Leica DMD 108 microscope from which micrographs at 4 × magnification of the whole cortical layer were obtained. Subsequently, they were examined with ImageJ v.1.51 in order to calculate the MCK density (MCKs count per mm^2^) and length (µm). MCKs were divided into two main categories according to their location within the cortex: osteonal or interstitial [15]. In cases that a MCK expands in both areas, the type was defined according to the most affected area.

The MCK density and length were evaluated in several cortical areas of 1 mm^2^ without overlapping MCKs.

For each area, the number of osteonal and interstitial MCKs was counted (MCKs/mm^2^) and the length of the MCKs was measured (µm). Furthermore, the proportion of osteonal MCKs in relation to the total MCKs (osteonal and interstitial) was calculated in each area.

### Statistical analysis

For descriptive statistics, the mean, median, standard deviation and quartiles were calculated. The normality of the variables was tested using the Shapiro Wilk test. Since none of the variables showed a normal distribution (*P* < 0.001), the non-parametric Mann–Whitney *U* test was used for comparative inferential analysis. The intra and inter-rater reliability of the data was analysed using the paired *T*-test and intraclass correlation coefficient (absolute agreement). For the intra-rater reliability, the observer who performed the data analysis (MWB) performed the MCK count a second time on a subsample. For the inter-rater reliability, this subsample was also analysed by a second observer (XJ). The statistical analyses were performed using Jamovi 1.0.4 and IBM SPSS Statistics v28.0.0. A significance level of 0.05 was set.

## Results

**Fig. 2:**
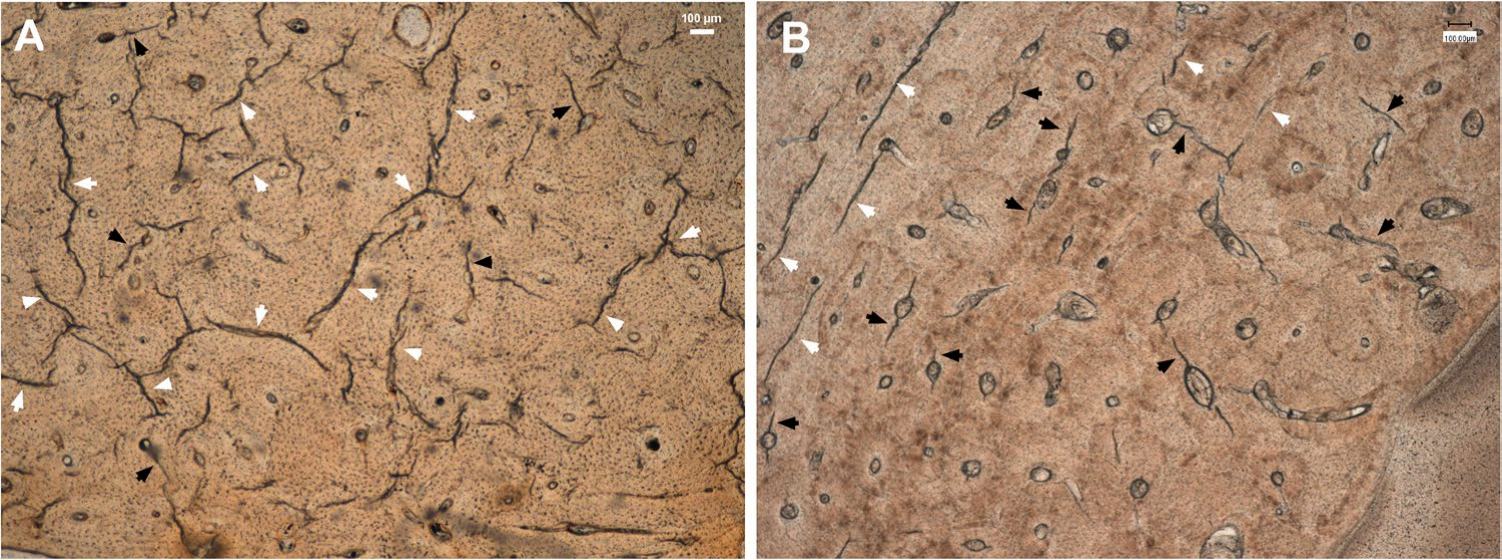
Micrographs of the histological sections of the studied fractured bones showing the microcracking pattern. Interstitial MCKs (white arrowheads). Osteonal MCKs (black arrowheads). **A** Dry bone with high density and greater length of interstitial MCKs. **B** Fresh bone with high density of short osteonal MCKs

**Fig. 3:**
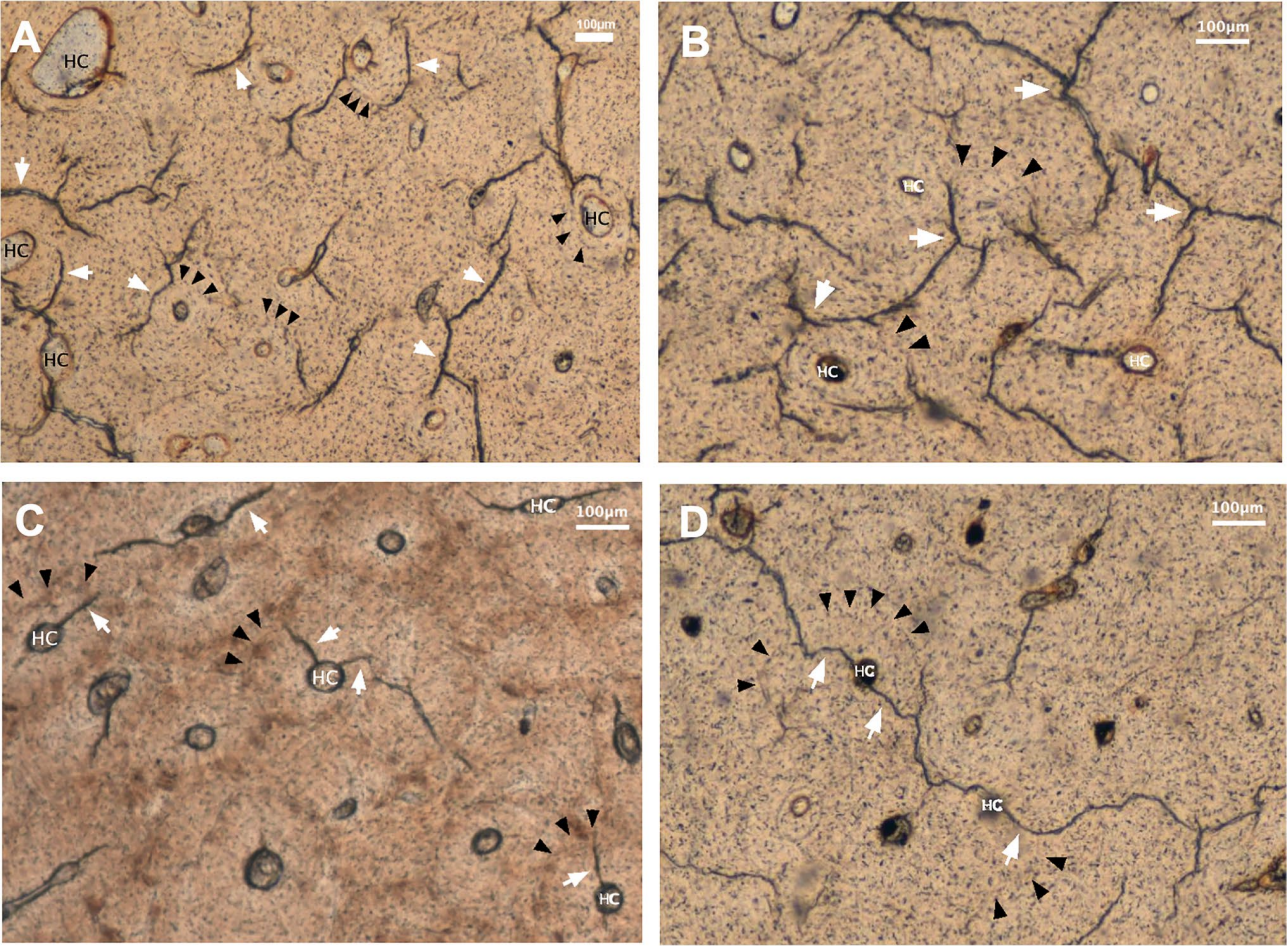
Micrographs of the histological sections of the studied fractured bones showing the microcracking pattern. MCKs (white arrowheads). Cement line delimiting Haversian systems (black arrowheads). Haversian canal (HC). **A**. Dry bone with MCKs following the direction of bone lamellas. **B**. Strongly branched long MCKs in the interstitial area of a dry bone that do not stop at the cement line. **C**. Short osteonal MCKs running from the Haversian canal to the cement line in a fresh bone. **D**. Long osteonal MCKs breaking a cement line in a dry bone

The microcracking pattern in both fresh and dry bone samples can be observed in Figs. 2 and 3. Table 1 shows the summary statistics of the MCK variables for the two sample groups of fractured bones.

**Table 1:** Descriptive statistics of MCK variables. *FB* fresh bones, *DB* dry bones. *N* the number of areas analysed for the MCK count, or the number of MCKs measured for length

MCK variables	Group	*N*	Mean	SD	Q1	Q2	Q3
Osteonal MCK density (per mm^2^)	FB	64	4.25	4.61	0.94	2.00	6.88
	DB	68	1.62	2.26	0.00	0.50	2.75
Interstitial MCK density (per mm^2^)	FB	64	3.85	2.86	1.25	3.50	5.75
	DB	68	9.64	9.32	1.75	4.63	15.80
Proportion (osteonal MCKs/interstitial MCKs) per mm^2^	FB	64	0.44	0.28	0.21	0.50	0.68
	DB	68	0.10	0.11	0.00	0.01	0.18
Osteonal MCK length (µm)	FB	995	106.95	92.32	47.89	78.66	134.96
	DB	303	108.02	90.79	41.35	77.41	145.31
Interstitial MCK length (µm)	FB	921	168.35	115.28	91.60	137.36	204.59
	DB	2155	181.23	115.68	98.30	150.40	230.61

MCK density in the osteonal area was higher in the autopsy—fresh—samples (x̅ = 4.25 ± 4.61 MCKs per mm^2^) than in dry specimens (x̅ = 1.62 ± 2.26 MCKs per mm^2^). Differences were statistically significant (Mann–Whitney *U* test; df 1313; *P* < 0.001). On the contrary to the osteons, the interstitial area of the dry specimens showed a higher MCK density (x̅ = 9.64 ± 9.32 MCKs per mm^2^) than the fresh samples (x̅ = 3.85 ± 2.86 MCKs per mm^2^). The differences were statistically significant (Mann–Whitney *U* test; df 1549; *P* = 0.004). The highest density of MCKs was found in the interstitial area of the dry bones, whilst the lowest is found in the osteonal zone of the same bones (Fig. 2A). The proportion of osteonal MCKs in relation to the total MCKs (osteonal and interstitial) was higher in the fresh specimens (x̅ = 0.44 ± 0.28) than the dry samples (x̅ = 0.10 ± 0.11) (Fig. 2B). The differences were statistically significant (Mann–Whitney *U* test; df 710; *P* < 0.001).

Regarding the MCK length, in the osteonal area there were no significant differences (Mann–Whitney *U* test; df 144,519; *P* = 0.683) between fresh (x̅ = 106.95 ± 92.32 µm) and dry samples (x̅ = 108.02 ± 90.79 µm). In the interstitial area, MCKs were shown to be longer than the osteonal ones (Fig. 3). Additionally, the longest interstitial MCKs were found in the dry specimens (x̅ = 181.23 ± 115.68 µm) (Fig. 3A, B and D) and the shortest in the fresh samples (x̅ = 168.35 ± 115.28 µm) (Fig. 3C). The differences were statistically significant (Mann–Whitney *U* test; df 898,609; *P* < 0.001).

The analysis of the control specimen (unfractured dry humerus) revealed that the amount of MCKs (x̅ = 0.21 ± 0.25 osteonal MCKs per mm^2^ and x̅ = 0.94 ± 0.68 interstitial MCKs per mm^2^) was significantly lower than in the dry fractured specimens (Table 1) (Mann–Whitney *U* test; df143; *P* < 0.001), suggesting that the histological technique was not contributing significantly to MCK propagation.

Table 2 shows the intra and inter-rater reliability analysis. The mean differences in MCKs count between observers (intra and inter) were statistically significant. Nevertheless, the intraclass correlation coefficient in all comparisons was high (*ICC* > 0.9), indicating very good intra and inter-rater agreement.

**Table 2:** Paired *T*-test and intraclass correlation coefficient *ICC* (absolute agreement) between raters. *OMCK* osteonal MCK count, *IMCK* interstitial MCK count, *MWB1* MWB first record, *MWB2* MWB second record, *XJ1* XJ first record

Paired *T*-test						ICC		
Raters comparison	Mean diff	SD	*T*-test	DF	*P*-value	Average measures	Lower 95%*CI*	Upper 95%*CI*
OMCK_MWB1 OMCK_MWB2	− 0.340	0.517	− 4.784	52	0.001	0.983	0.944	0.992
OMCK_MWB1 OMCK_XJ1	− 0.302	0.774	− 2.839	52	0.003	0.966	0.935	0.982
IMCK_MWB1 IMCK_MWB2	− 0.358	0.623	− 4.189	52	0.001	0.995	0.986	0.998
IMCK_MWB1 IMCK_XJ1	0.679	1.889	2.618	52	0.006	0.949	0.906	0.972

## Discussion

The current work uses a histological approach to assess whether there are differences in the microcracking pattern between perimortem and postmortem fractures. Specifically, we analysed the microcracking pattern in dry and fresh cortical bone, focusing on the density and the length of the MCKs in the osteonal and interstitial cortical area.

The results of our study showed that in both dry and fresh bone samples, MCKs were mainly located in the interstitial area following the direction of bone lamellas (Figs. 2A and 3A, B and D). This result is concordant with existing literature, which shows that 80–90% of all MCKs occur within interstitial bone or traverse the osteonal cement lines [16, 17]. In accordance with the existing literature, initiation, spreading and even accumulation of MCKs appear to be easier in the broad interstitial area [15, 18]. Jepsen et al. [18] emphasised that whilst the osteons have a rather low mineral content, the interstitial region exhibits enlarged hydroxyapatite crystals due to a lack of remodelling, resulting in an increased brittleness. In this context, brittle bone tissue is commonly known to be more likely to fracture [1–5]. Another variable that has been related to the formation and number of MCKs in the interstitial area is the density of osteocytes. According to Parfitt [19], the interstitial area has a lower density of osteocytes compared to the osteons. Qiu et al. [20] reported that the probability of MCKs in the interstitial region is five times higher than in the osteonal region due to this reduced interstitial osteocyte density. Likewise, Vashishth et al. [21] showed that a reduced osteocyte density is related to the accumulation of MCKs.

In our study, we generally observed a lower density of MCKs in fresh fractured bones (Fig. 2B). In this context, Mello et al. [22] histologically compared femoral bone samples from 20 exhumed individuals with a known period of burial between 6 and 15 years to fresh femurs from routine autopsy cases. They found that the mean osteocyte count was significantly higher in the fresh femurs than in the exhumed bone samples and interpreted the decreased osteocyte number as an indicator for bone degradation due to diagenetic processes. In contrast, collagen bundles were well preserved in exhumed bone and no difference was found compared to the fresh bone specimens. Thus, the assumption that the number of osteocytes limits the number of MCKs may be an explanation for our observation that the highest density and greatest length of MCKs were found in the interstitial area of dry bone. Consequently, our results raise the question of whether theses variables may be a marker to distinguish perimortem trauma from postmortem damage.

As a further potential marker for the fracture timing, we found that the proportion of osteonal MCKs in relation to the total MCKs was significantly higher in fresh fractured bones (on average, 44% of MCKs in 1 mm^2^ are located inside osteons) than in dry fractured bones (10%) (Fig. 2). The protective nature of osteocytes in the propagation of microdamage has been discussed previously. However, it remains unclear why more osteonal MCKs were found in the autopsy-fresh samples than in the dry fractured specimens. Several studies suggest that the osteocytes act as mechanical transductors. By means of the osteocyte canaliculi, they are able to sense their environment and react by inducing bone remodelling following stress or strain [17, 23–27]. Ebacher [11] observed intralamellar cracks and hypothesised that stress accumulation in the numerous canaliculi initiates microcracking as part of early bone damage detection and remodelling process. Since a reduced osteocyte density results in a decreased canaliculi density, their hypothesis is supported by the findings of Busse et al. [28], who described a deficient microdamage detection in older individuals due to a reduced osteocyte lacunar density. It is thus assumed that a reduced osteocyte network leads to failure and delay of remodelling, excessive MCK accumulation and increased bone fragility [11, 28]. Therefore, we suggest that our results in fresh perimortem fractures may be related to the mechanotransductor role of the osteocyte network in vital bone. This is clearly a point of the study that is not well understood and therefore needs more research.

Concerning the length of MCKs, the results showed that interstitial MCKs in fresh and dry bones mainly stop when they encounter a cement line (Fig. 3A). This finding has been experimentally shown by Boyce et al. [29], who observed that interstitial cracks are spreading between cement lines. Since the cement line is an area of reduced mineralisation, it provides a relatively ductile interface with the surrounding bone matrix [30]. According to this author, its elastic properties promote both, slow crack growth but also crack initiation. However, as it has already been reported by Boyce et al. [29], we also observed the longer and strongly branched MCKs do not stop at the cement line. This finding is particularly observed in dry bones (Fig. 3B).

O’Brien et al. [15] have already shown that the length of MCKs is actually the reason for the cement line to either function as a barrier or a weakness. The reason why osteons are stopping microdamage is that bone failure occurs with the propagation of one or two long cracks up to critical lengths and not with the convergence of several small MCKs. These authors reported that short MCKs around 100 µm stop at a cement line and MCKs over 300 µm are capable of crossing it.

Regarding the osteonal MCKs, we observed that they run from the osteonal canals to the cement line (Fig. 3C), and assuming that they initiate at the canal. This assumption would be in line with the hypothesis that void structures such as bone canals provide stress concentration sites for crack initiation, whilst bone lamellae and cement lines minimise the formation of larger cracks [11, 15, 31]. However, particularly in dry specimens, a minority of osteonal MCKs are long enough to break through the cement line (Fig. 3D). Here, the cement line is rather facilitating the crack propagation. These findings may explain why it has been claimed that osteons act as a weakness in bone and enhance crack propagation [32], whilst others reported that they act as barriers to further growth [15, 33].

In summary, this basic and exploratory study provides new insights into the characteristics of microfractures in the cortical tissue of fresh and dry bone as a result of blunt force trauma. Our results indicate a different pattern of microcracks between fresh and dry fractured bones. Specifically, the finding of a higher proportion of osteonal MKCs in fresh bone fractures is a promising finding for future research on histological differences between peri- and postmortem fractures. The reported differential microcracking pattern appears to be an original and important approach to explore differences between perimortem trauma and postmortem damage in the context of forensic anthropology.

### Limitations of the study

The small sample size of the study is the main limitation of the present work. As previously mentioned, this is an exploratory approach, and caution should be exercised when it comes to applying these results in the routine forensic fieldwork. Although the intra and inter-rater analysis showed an excellent agreement and indicates that the data are consistent, a better characterisation of the MKCs pattern in a bigger sample size would improve the reliability of the method. Moreover, future studies should take into account more variables such as bone variability and extrinsic factors, particularly those related to taphonomical conditions, as well as the experimental and the histological setting. This area clearly deserves further research in order to get a better understanding about microcrack production and propagation.
